# Zinc Status in Kurdish Adults With Hair Loss

**DOI:** 10.7759/cureus.67264

**Published:** 2024-08-20

**Authors:** Hivi Mahmoud, Payman M Saifdeen, Dhia Al-Timimi, Sedghi A Saeed

**Affiliations:** 1 Medical Chemistry, College of Medicine, University of Duhok, Duhok, IRQ; 2 Dermatology, Azadi General Teaching Hospital, Duhok, IRQ

**Keywords:** female pattern hair loss, male pattern hair loss, kurdish population, alopecia, hair loss, zinc status

## Abstract

Background: Zinc is an essential element for hair growth and may act as a strong inhibitor in accelerating follicle regression, besides being an accelerator for the recovery of the hair follicle. This study investigated the status of zinc in Kurdish adults with hair loss and its relation with each of the four types of hair loss.

Methods: We investigated the zinc status of a sample of Kurdish adults with hair loss who attended the Dermatology Outpatient Clinics at Azadi Teaching Hospital in Duhok, Kurdistan Region, Iraq. We included a total of 200 subjects in this study, of which 125 had hair loss with a diagnosis of alopecia areata, female pattern hair loss, male pattern hair loss, and telogen effluvium, and 75 were sex- and age-matched apparently healthy subjects without hair fall as a control group. Serum samples were used to measure zinc by colorimetric technique.

Results: In participants with hair loss, we found significantly lower serum zinc levels (p=0.002) compared with the control group. The telogen effluvium group had the lowest mean serum zinc level (p=0.006) and higher odds ratio compared with other hair loss groups (4.61). Overall, severe zinc deficiency was found in 12 (9.6%) subjects with hair loss, whereas none of the controls had severe zinc deficiency. Mild-to-moderate zinc deficiency was observed in 43 (34.4%) subjects with hair loss compared to one (1.3%) in the control group.

Conclusions: Our results showed that lower zinc status is linked to hair loss, especially alopecia areata and telogen effluvium in the Kurdish population.

## Introduction

Hair fall is considered one of the most common issues. It is a normal physiological condition that happens to everyone throughout life. A daily loss of 50-100 hairs is considered normal [1]. Hair loss is not recognized as a serious health problem but rather a cosmetic issue [2]. The main causes behind hair loss patterns generally are not fully understood [3]. In men, the male pattern hair loss (MPHL) is attributed mainly to a combination of genetic predisposition, and androgen hyperactivity that is mainly connected with the sensitivity of androgen action [4]. In women, female pattern hair loss (FPHL) may have a more complicated etiology. It is worth mentioning that, the action of genetic sensitivity to androgen combined indicates that it plays a pivotal role in many patients [5]. In addition, there are many other factors that may lead to hair loss including daily stress, pregnancy, and nutritional deficiency of some elements such as copper and zinc [6].

Zinc is one of the fundamental micronutrients that contributes significantly to the metallo-enzyme body process [7]. Zinc is involved in nucleic acid synthesis and protein, as well as in numerous cellular functions and metabolic actions [8]. Several studies have reported a potential link between nutritional deficiency of zinc and MPHL, FPHL, telogen effluvium (TE), and alopecia areata (AA) [6,9]. Another study has shown that zinc is a potent influencer of hair follicle regression, besides being an accelerator for the recovery of the hair follicle [10]. It is well known that zinc acts as a 5-alpha reductase inhibitor and blocks the creation of dihydrotestosterone (DHT) known as the main cause of baldness and hair fall [11]; thus, most prescribed medicines used to treat or prevent hair loss contain 5-alpha reductase inhibitors. Furthermore, it has been observed that zinc deficiency or insufficiency is a pathogenic hallmark in acrodermatitis enteropathica which is characterized by inflamed patches of dry red skin that then become crusted and blistered before revealing pustulent eroded lesions and eventually cause hair fall [12].

Studies on the link between zinc and hair loss have yielded controversial results; some have reported a significant relation between zinc deficiency and hair loss [13], others have highlighted no real relation [14], while yet others have reported that deficiency of zinc has a direct relation with TE as well as with MPHL [15]. However, to date, there are still arguments about zinc deficiency in hair loss, particularly among the Kurdish population, who are nutritionally at high risk of mild to moderate zinc deficiency [8,16]. Little is known about zinc status in hair fall cases, which prompted us to carry out this study.

## Materials and methods

Study population and study design

This was a case-control study conducted at Azadi Teaching Hospital in Duhok city, Kurdistan Region of Iraq, in 2019. The study was approved by the Ethics Committee at the Duhok Directorate of Health, Duhok, Iraq (approval number: 13052019-8). Verbal consent was obtained from all participants.

A retrospective sample was obtained using a random sampling procedure for patients with hair fall who attended the Dermatology Out-patient Clinic at Azadi Teaching Hospital between July and December 2019. A total of 200 subjects (aged 18-47 years) were included. Of these, 125 cases of hair loss were diagnosed with AA, FPHL, MPHL, and TE. The inclusion criteria were adult patients with hair loss who were not suffering from any secondary cause such as thyroid diseases (hypothyroidism), iron deficiency anemia, and vitamin D deficiency and did not take supplements containing elemental zinc for the last six months prior to the study. The exclusion criteria for patients were: age less than 18 years or more than 47 years, presence of any chronic diseases including renal, liver, and heart disease, recent history of infection, history of malignancy, pregnancy, history of chemotherapeutic drug use and positive history of using supplements containing elemental zinc within six months prior to sample collection. The remaining 75 were apparently healthy subjects who comprised the control group without any clinical evidence of hair fall. A pretested questionnaire was designed to collect information on age, gender, socioeconomic status, marital status, education levels, family history of the same condition, drug history, and history of any chronic diseases. nd

Body Mass Index (BMI) was calculated by dividing weight (kg) by squared height (m^2^) [8]. Five mL of blood was collected from each subject after an overnight fast at the Laboratory of Azadi General Teaching Hospital. The blood was placed in a VACUETTE® CAT plain tube (Greiner Bio-One, Kremsmünster, Austria), and then the serum was separated by centrifugation at 3000 rounds per minute for 10 minutes and stored at -28°C for later analysis of serum zinc. Serum zinc level was measured by colorimetric method using zinc kit (LTA S.R., Milan, Italy).

Categorization of hair loss

The categorization of subjects was performed according to the criteria given in Dermatology Essentials [17]. Subjects with hair loss were classified by specialist dermatologists into four main groups: (i) AA was defined as patches of non-scaring hair loss with exclamation mark hair, (ii) MPHL was defined as gradual conversion of terminal hairs into villus hairs in a highly reproducible pattern; MPHL is symmetric and progressive with some pattern variation, (iii) FPHL was defined as hair loss with Ludwig Scale; the most common pattern of FPHL is diffuse central thinning of the crown with preservation of the frontal hairline and often there is a frontal accentuation of the hair loss, creating a “Christmas tree” pattern, and (iv) TE was defined as increased hair shedding of otherwise normal telogen hairs.

Assessment of zinc status

Zinc status assessment based on serum zinc concentration: Severe zinc deficiency (zinc < 50 µg /dL), marginal deficiency (zinc 50-70 µg /dL), normozincemia (zinc level between 70 and 130 µg /dL), and hyperzincemia (zinc >130 µg /dL) [18].

Statistical analysis

We used IBM SPSS Statistics for Windows, Version 25.0 (Released 2017; IBM Corp., Armonk, New York, United States) to analyze the data. The laboratory parameter values were displayed as mean and standard deviation (SD). We used the chi-square test to compare variables and the student’s independent t-test to compare variables between groups. A p-value of <0.05 was considered a statistically significant cut-off value. To ascertain the relationship between hair fall groups, odds ratios and related 95% confidence intervals (CI) were used. Intergroup comparison was analyzed by one-way analysis of variance (ANOVA). 

## Results

Table 1 shows the general characteristics of the participants. A significant difference in serum zinc level was found between the hair loss and healthy control groups (p=0.002). No significant differences were found between the hair loss and healthy control groups with respect to age or BMI.

**Table 1:** General characteristic of study participants. * p-value is statistically significant

Characteristics	Patients (n=125), mean±SD	Controls (n=75), mean±SD	p-value*
Age (years)	27.5±10.2	26.0±9.9	0.31
Body mass index (kg/m^2^)	25.1±5.1	24.2±2.9	0.16
Zinc (µg/dl)	76.3±19.5	85.7±10.9	0.002

Table 2 shows serum zinc levels and the frequency distribution of each of the four types of hair loss among males and females. Overall, females comprised 68.0% and males 32.0% of the sample. The mean age was nearly similar in males compared to females. Fifty-three (62.3%) females had TE compared to six (15.0%) males (p= 0.001). Patients with AA were mostly males (45%) compared to females (11.7%) (p=0.001). The mean serum zinc level was lower in females compared to males (74.7±16.8 vs. 78.7±18.7 ug/dl), but the difference was not significant.

**Table 2:** Frequency distribution of hair loss types among males and females in the hair loss group (N=125) *p-value < 0.05 is statistically significant; data given as n (%) except in age and zinc, which are given as mean±SD AA: alopecia aerate; MPHL: male pattern hair loss; FPHL: female pattern hair loss; TE: telogen effluvium

Variable	Females (n=85), n (%)	Males (n=40), n (%)	p-value
Age (years), mean±SD	27.5±10.2	28.9±9.6	0.46
AA	10 (11.7)	18 (45.0)	0.001*
MPHL	0 (0.0)	16 (40.0)	-
FPHL	22 (25.8)	0 (0.0)	-
TE	53 (62.3)	6 (15.0)	0.001*
Serum zinc level (µg/dl), mean±SD	74.7±16.8	78.7±18.7	0.40

Table 3 shows the frequency distribution of zinc status among subjects with hair loss and control subjects. According to the zinc status distribution analysis, patients with AA and TE had higher frequencies of severe zinc deficiency (14.3% and 11.8%, respectively) than those with MPHL and FPHL (0.0% and (0.0%), whereas none of the controls had severe zinc deficiency. Mild-to-moderate zinc deficiency was observed in 54 (43.2%) patients with hair loss compared to one (1.33%) of the control subjects.

**Table 3:** Zinc status in patients with hair loss and control subjects AA: alopecia aerate; FPHL: female pattern hair loss; MPHL: male pattern hair loss; TE: telogen effluvium

Zinc (µg/dl)	AA (n=28), n (%)	MPHL (n=16), n (%)	FPHL (n=22), n (%)	TE (n=59), n (%)	Hair Loss Group total (n=125), n(%)	Control Group total (n=75), n (%)
<50	4 (14.3)	0 (0.0)	0 (0)	7 (11.8)	11 ( 8.8)	0 (0)
50-70	11 (39.3)	8 (50.0)	9 (40.9)	26 (44.1)	54 (43.2)	1 (1.33)
>70	13 (46.4)	8 (50.0)	13 (59.1)	26 (44.1)	60 (48.0)	74 (98.66)

To determine which of the four types of hair loss was significantly associated with serum zinc level, the patients were divided into two groups based on the lower normal limit of serum zinc level (70 ug/dl), as shown in Table 4. We found a significant difference in the low zinc group between those with TE and other types of hair loss ( p=0.006). Regarding OR, the TE group had significantly higher OR compared to the other groups (4.61, P=0.010).

**Table 4:** Patients with low and normal serum zinc levels *p-value is statistically significant AA: alopecia aerate; MPHL: male pattern hair loss; FPHL: female pattern hair loss; TE: telogen effluvium

Type of hair fall	Patients having zinc <70 ug/dl (n=65), n (%)	Zinc levels (ug/dl), mean + SD	Patients having zinc >70 ug/dln (n=60), (%)	Zinc levels (ug/dl), mean + SD	Unadjusted OR (95%Cl)
AA	13 (20.0)	54.4±11.4	15 (25.0)	83.9±13.3	2.95 (1.27-9.01)
MPHL	6 (9.2)	64.2±5.8	10 (16.7)	88.8±10.4	1.65 (0.71-7.7)
FPHL	4 (6.2)	64.4±4.6	18 (30.0)	76.9±7.5	0.56 (0.24-3.0)
TE	42 (64.6)	48.6±9.4	17 (28.3)	86.6±11.2	4.61 (1.91-9.73)
P-value		0.006*		0.086	0.010*

## Discussion

Hair loss is a common problem nowadays. Studies have reported a potential link between nutritional zinc deficiency and MPHL, FPHL, TE, and AA [9,13,19]. To our knowledge, this is the first prospective investigation of the association between zinc status and hair loss problems in the Kurdish population in Duhok city, Iraq. The current study showed a significant difference in zinc levels between the hair loss and healthy control groups. The results confirmed an association between AA and severe zinc deficiency, and a similar result was observed for TE. These results are agreed with previous studies [20,21].

The low zinc status was most frequent in patients with TE, as compared with other groups, indicating an important mechanism through which zinc can influence a higher risk of hair loss in the Kurdish population. The results of the present study showed that two-thirds of females had TE, while nearly half of the males had AA. It was noticeable that approximately one-half of the patients in this study had moderate to severe zinc deficiency, and it was more prevalent among patients with AA and TE, whereas none of the controls had severe zinc deficiency. Mild to moderate zinc deficiency exists in several countries, specifically developing countries [18], in light of the fact that the regularly utilized nourishment has low zinc content and is rich in phytate. The phytate content of grain protein is known to diminish the accessibility of zinc. Along these lines, the prevalence of zinc deficiency is probably going to be high in a population expending enormous amounts of oat/cereal proteins [21]. These adjustments in zinc status and increment in hair loss are regularly clarified by inadequacies coexistence of zinc because of normal dietary sources and less absorption in intestines by similar dietary factors [18].

Zinc has been shown to have an anti-androgenic effect and it specifically inhibits 5α-reductase type 1 and 2 activity, thus promoting hair growth. It was also seen to have a considerable effect on hair growth in androgenic alopecia in a randomized clinical study [22].

Several studies have demonstrated that serum zinc levels are low in AA patients [23,24], but the pathogenesis of this decrease is not well known. As cofactors of metallo-enzymes, zinc affects almost all parts of the metabolism considerably affecting the organs of the body, including the skin. Acquired and congenital zinc deficiency is normally communicated as a type of skin condition, for example, acrodermatitis enteropathica, psoriasis-like blisters, eruptions, onychopathy, and hair fall [12]. It has been shown that hair improved through the oral administration of zinc compounds [25]. However, a study by Cousins et al. revealed that zinc compounds taken orally as supplements had no remedial impact on hair loss [26].

Hair loss is difficult to ignore, common, and stressful, provoking anxieties and more intense than its objective severity seems. The burden of hair loss for some patients may be comparable to more severe chronic or life-threatening diseases [27]. The problem of hair fall is an important part of dermatology, and it is expanding its scope gradually. Depending on the findings of this study, it is recommended to use serum zinc measurement, and zinc supplementation if the concentration of zinc is lower than the cut-off values for assessment of zinc deficiency. Even though the current study did not measure the concentration of zinc in the hair itself, big-scale studies on the concentration of zinc in both serum and hair are needed for zinc supplementation in the future. Apart from zinc and micronutrient deficiency, there are other factors that might affect hair loss such as genetics, age progress, smoking, hormones, and the kind of soaps and shampoos used [11,13,19], all these factors should be taken into consideration before final conclusion. 

Limitations

Other relevant minerals related to hair fall mechanism were not measured and this is the main limitation of the study; this might undermine the definitive conclusion regarding the role of zinc specifically related to these patients with hair loss. The inability to utilize dietary assessment methods such as food records to capture detailed dietary patterns is another limitation of the study. Not measuring hair zinc level was a major limitation of the study which may undermine the whole picture of the association between zinc level and hair loss. Despite these limitations, the strength of the current study was that it showed a strong association between zinc deficiency and some types of hair loss.

## Conclusions

Our results indicated that hair loss characterized by AA and TE may be related to zinc deficiency in the Kurdish population. Furthermore, we found that approximately one-half of the patients with hair loss were with moderate-to-severe zinc deficiency. Therefore, routine screening of serum zinc and zinc supplementation may be of great importance.
